# Geographic Variation in Loneliness and Social Isolation in Australia: Socio-Demographic and Healthcare Utilisation Determinants

**DOI:** 10.3390/healthcare14101318

**Published:** 2026-05-12

**Authors:** Arul Earnest, Michelle H. Lim, Lidia Engel, Kate Filia, Sharon Clifford, Fikru Rizal, Laura Hayes, Sophia Zoungas, Ahmadreza Pourghaderi, Hossein Nejati, Michael Berk, Long Khanh-Dao Le, Helen Skouteris, Cathrine Mihalopoulos

**Affiliations:** 1 Biostatistics Unit, School of Public Health and Preventive Medicine, Monash University, Melbourne, VIC 3004, Australiasharon.clifford@monash.edu (S.C.); sophia.zoungas@monash.edu (S.Z.);; 2 Faculty of Medicine and Health, School of Public Health, The University of Sydney, Sydney, NSW 2050, Australia; 3 Orygen Youth Health, Parkville, VIC 3052, Australia; kate.filia@orygen.org.au; 4 Mind Australia, Burnley, VIC 3121, Australia; 5 School of Medicine, Deakin University, Geelong, VIC 3125, Australia

**Keywords:** loneliness, social isolation, geospatial, Bayesian analysis, socio-economic, healthcare utilisation, public health

## Abstract

**Background:** Loneliness and social isolation are major public health challenges linked to premature mortality and significant healthcare and productivity costs. However, their geographic distribution and socio-demographic determinants remain poorly understood, with few studies applying spatial methods to identify high-need areas and protective factors. **Methods:** This study aimed to investigate the geographic distribution and determinants of loneliness and social isolation across Australia using a spatial epidemiological approach. Utilising longitudinal data from the Household, Income and Labour Dynamics in Australia (HILDA) survey, along with Australian Bureau of Statistics (ABS) census data, greenness vegetation index and walkability index, we employed Bayesian conditional autoregressive (CAR) models to assess spatial and temporal patterns at the Statistical Area Level 3 (SA3) over a 22-year period and present the relative risks (RR) and credible intervals (CrI). **Results:** Our analysis revealed spatial variation in the RR of both loneliness and social isolation, with notable hotspots in socioeconomically disadvantaged areas. In multivariable models, area-level socio-economic disadvantage (as measured by the Index of Relative Socio-economic Advantage and Disadvantage, IRSAD) (RR = 0.8, 95% CrI: 0.76–0.85) for the highest quintile (most advantaged) and a higher prevalence of depression and/or anxiety (RR = 4.3, 95% CrI: 3.0–6.1) were associated independently with relative risk of loneliness but not with age structure, remoteness, green space or walkability index. For social isolation, higher average hospital admission rates per region were the strongest factor. **Conclusions:** The spatial heterogeneity observed in our study underscores the need for place-based public health responses, including community-based interventions and targeted resource allocation, especially in disadvantaged communities.

## 1. Introduction

Loneliness and social isolation have emerged as critical public health challenges. They are associated with adverse outcomes, including cardiometabolic disease, stroke, dementia, depression and interact with other risk factors (e.g., age, gender, socioeconomic status, employment and migrant population status) summarised in a recent study [1]. Loneliness significantly increases the risk of premature mortality by 26%, comparable to other major risk factors such as obesity and smoking, highlighting its relative critical role as a public health concern [2]. Based on a recent comprehensive systematic review on the economic costs of loneliness and social isolation, an estimated excess cost of USD 2 billion (Australia) to USD 25.2 billion (Spain) per year is projected for healthcare expenses and lost productivity, compared to individuals who are not affected by loneliness and social isolation [3].

While individual level studies have evaluated the socio-demographic, physical and mental health determinants of loneliness and social isolation [4], the analysis methods were non-spatial and did not account for clustering of cases within geography, and the results cannot be generalised to the whole of Australia. [5]. Similarly, while loneliness and social isolation have been examined in the health geography literature [5,6] using neighbourhood and spatially informed approaches [7,8], comparatively fewer studies have applied national-scale spatial–temporal models that explicitly account for spatial dependence and small-area instability when estimating area-level burden. It is important to consider such spatial techniques in order to find areas of particular need, whereby the delivery of interventions that can address loneliness and social isolation can be prioritised. Exploration of geographical distributional differences can help uncover protective factors of particular areas that can help inform a broader approach of addressing higher levels of loneliness or social isolation.

Loneliness in later life is increasingly recognised as a significant public health concern, with growing evidence linking it to adverse physical and mental health outcomes through behavioural, psychosocial, and physiological pathways. For example, Green et. al. [9] highlight how loneliness appears to operate through a pathway in which increased depressive symptoms lead to poorer self-rated health, ultimately contributing to a higher burden of chronic conditions. A review of longitudinal studies by Dahlberg and colleagues [10] suggests that loneliness in later life is shaped by a relatively small set of consistent risk factors, including partner loss, social isolation, low activity levels, poor self-rated health, and depression. In addition to individual-level determinants, Victor et. al. [11] report that older adults living in more socioeconomically deprived areas experience higher levels of loneliness independent of individual-level characteristics, highlighting the importance of area-level deprivation in shaping loneliness risk trajectories.

Our study is informed by the concept of opportunity structures, a framework articulated within Australian public health and health geography literature, which emphasises how place-based social, economic, and physical environments shape opportunities for health, participation, and social connection. Early work by Baum and Palmer [12] highlighted the possibility of how neighbourhood-level resources, infrastructure, and socio-economic conditions can enable or constrain individual behaviours and social engagement, independent of individual characteristics. Within this framework, factors such as area-level socio-economic disadvantage, access to services, walkability, green space, and healthcare infrastructure can be understood as structural opportunities/constraints, that influence the likelihood of social interaction and the experience of loneliness or social isolation.

In addition to contextual socio-demographic status, some studies have considered the association between other environmental measures such as vegetation cover, as defined by the Normalised Difference Vegetation Index (NDVI), green space and other health outcomes, with mixed study design methodologies and conflicting results. Green space and tree canopy cover reduced the incidence and prevalence of social isolation (moderating effect by sex), in a study of people aged 45 and up in New South Wales [13]. The study accounted for clustering of people at the geographic level, but not geographic correlation between regions. NDVI has also been found to be positively associated with food allergy (i.e., greater greenness with higher rates of allergy), with a moderating effect through socio-economic status, in a study restricted to Melbourne, Australia [14]; however, the analytical method was non-spatial. Zhou et al. found a protective effect of NDVI on mortality across Australia using an age–cohort–period study [15], while a third non-spatial study [16] found a positive effect of NDVI on the subjective well-being of subjects in Victoria, Australia, with the magnitude of relationship increasing with neighbourhood size. To the best of our knowledge, NDVI has not been studied with loneliness or social isolation.

Statistical techniques to analyse geospatial data have matured over the last decade, mainly due to the availability of good quality, high resolution population level data and digital maps with boundaries, particularly in developed countries including Australia. Coupled with this, advancements in computing speed and facilities have enabled the adoption of Bayesian spatial analytical techniques to study diverse populations and outcomes, including for example patient-reported quality of life among prostate cancer patients [17], surgical delay among non-small cell lung cancer patients [18], out-of-hospital cardiac arrests [19] and diabetic emergencies attended by prehospital Emergency Medical Services [20]. These Bayesian analytical techniques allow improved estimation of relative risks in geographical data, through “borrowing strength” from neighbouring regions, particularly when the expected counts are small [21].

This research directly addresses priorities outlined in the Ending Loneliness Together White Paper [2], which emphasises strengthening the Australian evidence base on loneliness and accelerating the translation of research into policy and practice. By generating robust, localised data on the distribution and determinants of loneliness, this study contributes new, context-specific evidence for Australia. In this study, we aim to provide a distinct, national-scale spatial analysis of loneliness and social isolation in Australia. Specifically, the objectives are as follows:

(1) To quantify the spatial and temporal variation in loneliness and social isolation across Statistical Area Level 3 (SA3) regions;
(2) To identify and compare area-level socio-demographic, environmental, and healthcare utilisation factors associated with each outcome;
(3) To apply Bayesian spatial–temporal models to generate stable small-area estimates that account for spatial dependence and heterogeneity.

By integrating longitudinal health data with aggregate spatial covariates, this study provides a comprehensive and methodologically robust assessment of the geographic distribution and determinants of loneliness and social isolation in Australia.

## 2. Methods

### 2.1. Data Sources

Data were sourced from the Household, Income and Labour Dynamics in Australia (HILDA) Survey, Release 22 [22], a nationally representative longitudinal study that began in 2001. In its initial wave, HILDA gathered information from approximately 13,000 individuals in 7000 households using a combination of self-completed questionnaires, in-person interviews, and telephone interviews with participants aged 15 years and older. These interviews provided rich individual-level measurements of psychosocial constructs, including loneliness and social isolation. The HILDA Survey uses a mixed-mode data collection approach, including interviewer-administered interviews (primarily face-to-face, with telephone interviews where necessary) and a self-completion questionnaire administered in paper or online formats. The choice of mode is driven by operational considerations rather than geographic or participant-specific criteria [23]. Complementary geographic and demographic information was sourced from the Australian Bureau of Statistics (ABS) 2021 Census data [24]. The merging of these datasets at an aggregate spatial level allows for a robust spatial–temporal analysis of psychosocial outcomes across Australia. This study was conducted in accordance with the Declaration of Helsinki and approved by the Monash University Human Research Ethics Committee (approved project ID: 42237).

### 2.2. Outcomes and Exposure Variables

Loneliness was assessed using a 1–7 agreement scale with 1 being “strongly disagree” and 7 being “strongly agree”, and individuals classified as experiencing loneliness if they scored ≥5 in response to the statement, “*I often feel very lonely*” [25]. Social isolation was operationalised as a composite measure derived from multiple self-reported indicators within the HILDA survey, including frequency of social contact with friends or relatives, participation in social or community activities, and household living arrangements. Individuals were operationally classified as socially isolated if they lived alone and reported social contact outside the household less than once per month (taken from the scale: 1. Every day, 2. Several times a week, 3. About once a week, 4. About once every 2–3 weeks, 5. About once a month, and 6. Less often than once a month). In our analysis, we also chose to examine episodic loneliness and social isolation (i.e., the number of episodes of outcomes over the years where data was collected for that respondent). Episodes were defined at the individual level based on observed survey responses within each wave, with individuals able to contribute multiple episodes across waves. These individual-level episodes were then aggregated to the corresponding SA3 and survey period counts, with the corresponding denominator defined as the total number of person-years contributed within each SA3/survey period. Participants contributed person-time only for waves in which they were observed, and missing waves were handled by using available observations (complete case analysis without imputation. Survey weights were not applied, as the analysis was ecological in nature and focused on estimating relative spatial risks and geographic variation rather than population-level prevalence.

In addition to loneliness and social isolation, the following variables were also computed from HILDA: the proportion of individuals with self-reported diagnoses of any physical chronic/serious illness diagnoses, depression and/or anxiety, long term health conditions/disability (e.g., sight problems, hearing problems, speech problems, difficulty in learning or understanding things) and average number of self-reported medical (GP or Specialists) doctor visits and number of hospital admissions in the past 12 months.

The Index of Relative Socio-economic Advantage and Disadvantage (IRSAD) was used to rank areas using weighted combinations of selected variables and is best interpreted as an ordinal measure [24]. Applied to geographic areas, not individuals, it reflects the collective socio-economic conditions of residents using data aligned with the Australian Statistical Geography Standard (ASGS) Edition 3 [26]. Higher values across the 5 levels of quintiles indicate a greater degree of advantage in that region compared to other Statistical Area Level 3s (SA3s). Remoteness Index [27]: Area-level remoteness was classified using the Accessibility/Remoteness Index of Australia Plus (ARIA+), which categorises regions based on their relative access to services such as healthcare, education, and retail. SA3s were grouped into major cities, regional areas (inner and outer regional), and remote areas (including very remote), based on standard ARIA+ classifications [28]. Additionally, we included the geographic proportions within each spatial unit for the following demographic characteristics: individuals aged over 65 years (approximate retirement age pension in Australia), male sex, Australian-born persons, Aboriginal and Torres Strait Islander individuals from the ABS census 2021 data. These variables were included as prior research [5,6] has shown these factors to be associated with increased risk of loneliness and social isolation. These were left as continuous variables and can be interpreted as percentage-point changes at the SA3 level from the regression coefficients, for ease of interpretability.

Greenspace quantification was conducted at the Statistical Area Level 3 (SA3) across Australia using the Normalized Difference Vegetation Index (NDVI) derived from the MODIS MOD13Q1 satellite data [29]. MOD13Q1 delivers 16-day composite NDVI data at a 250-metre spatial resolution and has been extensively validated worldwide for its accuracy in capturing vegetation cover. NDVI ranges between negative (water) to positive (very green), with 0 indicating minimal vegetation. We grouped NDVI into quartiles. NDVI was used to approximate area-level greenness, focusing on the quantity (density) and health of green vegetation in an area.

The walkability index is a composite measure of how conducive an area is to walking for daily needs such as accessing shops, services, green spaces, and public transport [30]. It is a key urban liveability indicator used to assess physical activity potential and transport accessibility. In this study, walkability scores were obtained from the Australian National Liveability Dataset (ANLD), which includes standardised spatial indicators across Australian cities at small-area levels (e.g., SA3) [31,32]. The index integrates multiple components reflecting the built environment: residential density, street connectivity (e.g., intersection density), land use mix (proximity to amenities), and access to public transport and recreational spaces. We grouped the index into quartiles, with higher values denoting more walkable, amenity-rich, and connected neighbourhoods, and negative values indicating low-density, car-dependent, and poorly connected areas. The provenance and harmonisation of covariates at the SA3 level is shown in Table S1.

### 2.3. Spatial and Temporal Aggregation

There are 358 SA3 regions in Australia. Non-resident and “no usual address” comprise 18 regions and were excluded from the analysis. From the remaining 340 SA3s, after excluding 5 regions that were remote regions not sampled by the HILDA study (Cocos Island, Blue Mountains South, Christmas Island, Jervis Bay and Norfolk Island), 335 discrete regions across Australia remained [26]. SA3s generally have populations ranging from 30,000 to 130,000 people. In regional areas, they typically cover the catchment areas of regional cities with populations exceeding 20,000. In major cities, SA3s correspond to areas served by key transport and commercial hubs. SA3s are broadly comparable in scale to U.S. counties or groups of census tracts. Although other geographical boundaries exist, SA3 was chosen as the optimal geography to provide our study with a greater precision in parameter estimates, while maintaining data confidentiality. SA3 is also the most appropriate geography of choice for health atlases in Australia, as we observe from the Australian atlas of healthcare variation, which geographically displays various chronic disease, infection and surgical data [27].

SA2 is an alternative level of geography, but the low event rates, particularly in regional areas, may not provide the required precision to reliably estimate and report the relative risks with confidence. In contrast, SA4 regions are substantially larger, typically encompassing populations of far more than 100,000 residents, and may therefore obscure meaningful within-region heterogeneity relevant to place-based health planning and intervention.

To improve the precision of parameter estimates while retaining meaningful temporal resolution, data were aggregated into three equal multi-wave intervals aligned with the HILDA survey structure: Period 1 (Waves 1–7; 2001–2007), Period 2 (Waves 8–14; 2008–2014), and Period 3 (Waves 15–22; 2015–2022). Aggregation across waves within each period increased the stability of SA3 level estimates by mitigating sparse event counts, particularly in less populous regions. For each SA3 period, pooled counts and corresponding expected values for both exposure and outcome variables were derived, with total person-years contributed serving as the denominator. This combined geographic and temporal aggregation enabled robust estimation of spatial patterns and temporal trends in loneliness and social isolation, while accounting for heterogeneity across communities and over time. Supplementary Table S1 explicitly describes the provenance, reference years, and harmonisation of all covariates (time-invariant) used in the analysis.

### 2.4. Statistical Model

A Bayesian spatial–temporal modelling framework [33] was implemented to explore the association between the various socio-demographic, health utilisation variables and the outcomes. Specifically, Bayesian conditional autoregressive (CAR) models [34] were employed to partition the random variation into spatially structured and unstructured components. As for spatial dependency, the spatial structure was defined using a Queen’s contiguity-based adjacency matrix, which recognises neighbouring SA3 regions based on shared boundaries or vertices. This approach effectively allows for the “borrowing of strength” from adjacent areas to yield more precise smoothed relative risk estimates. Geographically isolated SA3s with no contiguous neighbours were retained in the analysis and treated as having no adjacent regions, allowing their risks to be estimated independently while remaining within the overall spatial framework.

The observed outcome follows a Poisson model as shown below:

O_ik_ ~ Poi(µ_ik_)

log(µ)_ik_ = log(E_ik_) + u_i_ + v_i_ + β_1_ × t + β_2_ × t^2^ + x_ik_′β

where O_ik_ and E_ik_ are the observed and expected counts of loneliness (or social isolation) for the ith SA3 (i = 1, 2, …, 335) and kth time period (k = 1, 2, 3), u_i_ is a spatially structured random effect and v_i_ is the spatially unstructured random effect term, β_1_ and β_2_ are coefficients for time and a quadratic term in time, and β represents a vector of effect sizes for the covariates, respectively. The observed counts correspond to episodic occurrences of the outcomes. The expected counts are obtained via indirect standardisation, calculated by applying the overall Australian outcome rates to the population structure of each SA3, thereby representing the number of cases expected in each area if it experienced the national average rate. The relative risk (RR) estimates are defined as the ratio of observed to expected counts. An RR greater than 1 indicates that the risk of loneliness (or social isolation) in a given area is higher than the overall Australian average, while an RR less than 1 indicates a lower-than-average risk. The Poisson model is appropriate as the outcomes are counts in nature. For the spatially structured term, we utilised the conditional autoregressive (CAR) model:

[u*_i_*|u*_j_*, *i* ≠ *j*, τ_u_^2^] ~ N (ū*_i_*_,_ τ*_i_*^2^),

where ${ū}_{i}=\pi \frac{1}{\sum_{j}w_{ij}}\sum u_{j}w_{ij}$

$${\tau_{i}}^{2}=\frac{{\tau_{u}}^{2}}{\sum_{j}w_{ij}}$$

Here, w_ij_ = 1 *if SA3 regions i* and *j* are adjacent, and 0 otherwise. The Queen neighbourhood adjacency matrix was defined, which included all immediate neighbours that shared a common border. Each neighbour contributed equally to the adjacency weight matrix (*weight, w = 1*). For the spatially unstructured random effect term and all the fixed effects, the means were assigned non-informative normal distribution with mean 0 and hyperparameter precision term (i.e., standard deviation~*Uniform(0.001,10)*). The spatially structured term followed a CAR normal model as above, with a similar non-informative precision term.

For each model, we ran two different Markov chain Monte Carlo (MCMC) chains, with diverse initial values (total of 700,000 iterations per chain). We discarded the first 300,000 iterations as burn-ins and used the remaining 400,000 iterations to calculate the posterior distribution of the parameters. We also undertook a thinning interval of 2 to minimise autocorrelation observed among samples.

Convergence of the MCMC chains was rigorously assessed using the Gelman–Rubin diagnostic tool, ensuring that the posterior distributions stabilised before inferences were drawn. From the results of the univariable analysis, starting from the most significant covariate, we used the forward variable selection method and built a parsimonious multivariable model with all resulting covariates that were significant. RR and their 95% credible intervals (CrI) were calculated for each covariate as measures of effect size, with model adequacy indicated by the CrI not including the null value of 1. We present the RRs for the most recent time period (2015–2022) as the most relevant for current policy decision-making.

Data preparation and cleaning were undertaken in Stata (V18.3) and the Bayesian analysis performed in OpenBUGs software (V3.2.3).

## 3. Results

This study included 335 SA3s representing 454,748 subject episodes across 22 waves. The pooled episodic prevalence of loneliness was 18.1% (54,385/301,342), while the prevalence of social isolation was 5.1% (15,139/299,877). Table 1 provides a description of demographics and health utilisation patterns across all waves of the HILDA Survey in Australia. Overall, 71% of people were Australian born, but this varied at the SA3 level between 33% and 87%. Fifteen percent of the individuals across all regions reported depression and/or anxiety (range 0–50%). The average number of doctor visits was 4.8 (range 1–11) while the mean number of hospital admissions was 0.21 (range 0–0.8). The Normalized Difference Vegetation Index (NDVI) ranged from 0.16 to 0.74. These results highlight the spatial variation in the covariates at the SA3 level.

Table 2 identifies the univariable covariates associated with geographic variation in loneliness. IRSAD, proportion: any chronic/serious illness, depression and/or anxiety and average number of doctor visits and hospital admissions were all found to be associated significantly with the outcomes. There was a dose–response relationship between IRSAD and loneliness, for example, those living in quintile 5 (areas of most advantage) were 25% less likely to report feelings of loneliness (95% CrI: 20–29%) as compared to quintile 1 (areas of most disadvantage). The risk of loneliness increased by a factor of 6.1 (95% CrI: 4.1–9.0) for every percentage point increase in the proportion of depression and/or anxiety in a region. Areas with higher levels of walkability had lower risk of loneliness (e.g., RR = 0.89 for the fourth quartile compared to first quartile). Age, remoteness, NDVI and proportion with long term health conditions were not associated with areal variation in risk of loneliness. Figures in Table 2 shown in bold indicate statistical significance.

Similarly for social isolation, univariable analysis is presented in Table 3. IRSAD (quintile 4), remoteness (regional areas), NDVI, proportion of depression and/or anxiety along with long term health conditions and the average number of doctor visits, hospital admissions and walkability index were identified as significant contextual factors. Those living in regional (inner and outer region) areas were 1.2 (95% CrI: 1.0–1.4%) times more likely to have social isolation compared to those living in metropolitan areas. The areal risk of social isolation increased by a factor of 3.7 (95%CrI: 2.1–6.6%) for each unit increase in average number of hospitalisations across SA3s.

In the multivariable model (Table 4), only IRSAD and proportion with depression and anxiety were significantly and independently associated with loneliness at the SA3 level. Those living in quintile 5 (more advantaged) were less likely to report loneliness (RR = 0.80, 95% CrI: 0.76–0.85) as compared to those living in quintile 1 (more disadvantaged). After accounting for IRSAD, the risk of loneliness increased by a factor of 4.3 (95% CrI: 3.0–6.0%) for each unit percentage change in the proportion of people with depression and anxiety between SA3 regions. As for social isolation, only the number of hospital admissions remained significant, indicating it was the strongest contextual factor among all the variables.

**Figure 1:**
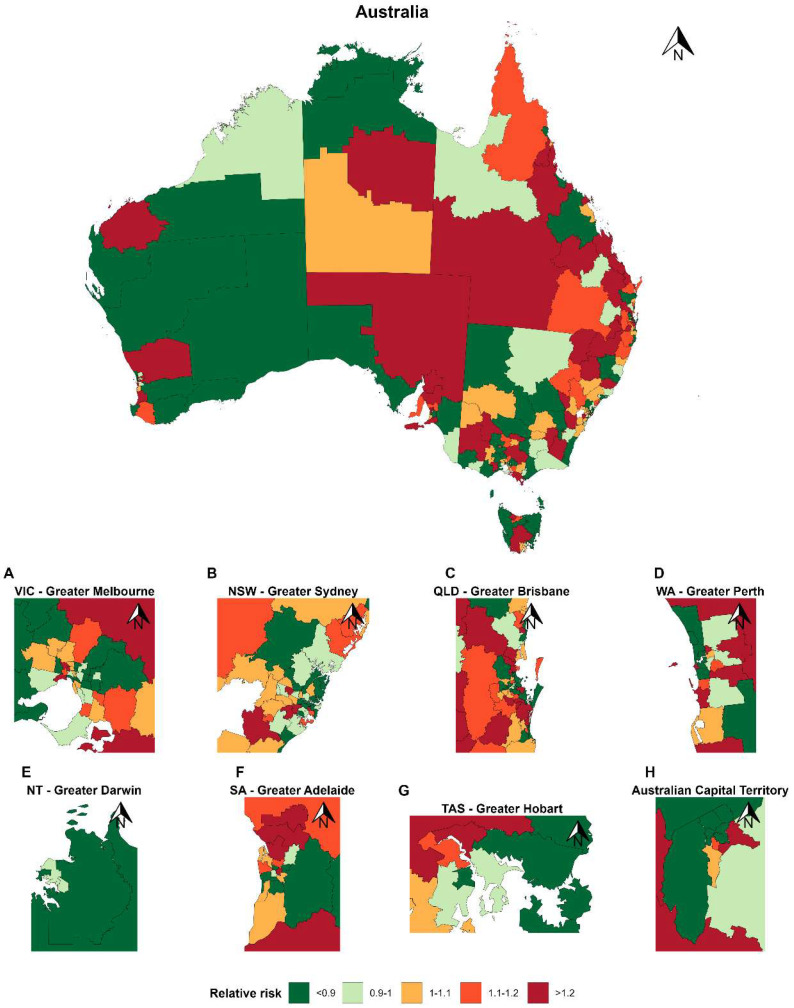
Distribution of relative risk of loneliness across Australia (SA3) in the period 2015–2022. Geographic variation in loneliness across SA3 regions in Australia. SA3s are medium-sized areas covering both metropolitan and regional populations. Values represent relative risk estimates, with red shades reflecting higher levels and green lower values). Higher levels are observed in socio-economically disadvantaged areas and regional areas.

**Figure 2:**
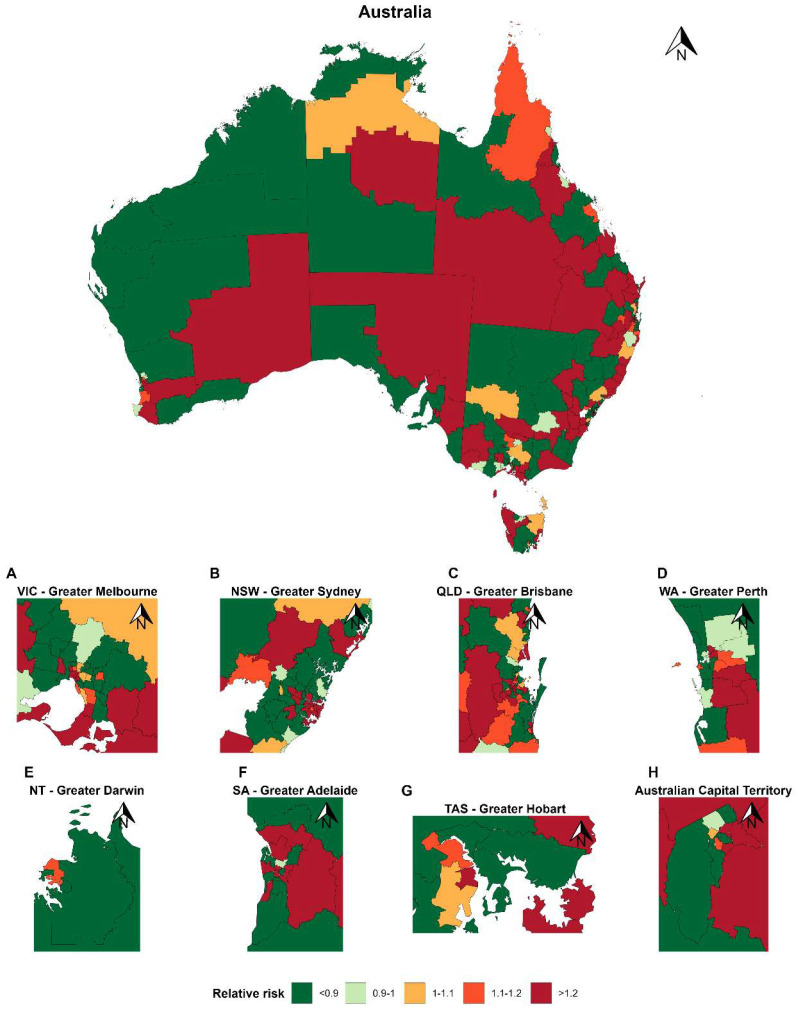
Distribution of relative risk of social isolation across Australia (SA3) in the period 2015–2022. Geographic variation in social isolation across SA3 regions in Australia. SA3s are medium-sized areas covering both metropolitan and regional populations. Values represent relative risk estimates, with red shades reflecting higher levels and green lower values. Geographic heterogeneity is seen across regions within each State.

The spatial distribution of loneliness and social isolation across Australia from 2015–2022 is shown in Figure 1 and Figure 2. Table S2 shows evidence of meaningful spatial clustering for loneliness, with an estimated 58.2% of the total residual variation attributable to spatially structured effects, while social isolation exhibited lesser spatial dependence (14.7% of variation attributable to spatial effects), suggesting that its remaining variability was driven predominantly by spatially unstructured heterogeneity rather than consistent geographic clustering. The spatial distribution of loneliness and social isolation reveals clear and interpretable patterns across Australia. Several SA3s demonstrate persistently elevated relative risk across time periods, forming stable hotspots predominantly in socioeconomically disadvantaged metropolitan fringe and selected regional areas. In contrast, lower-risk clusters are consistently observed in inner metropolitan regions. Maps for earlier time periods 2001–2007 and 2008–2014 are presented in Supplementary Figures S1 and S2, and indicate that while localised fluctuations are evident, the broader spatial distribution remains relatively stable across study years.

Model diagnostics indicated satisfactory convergence of the Markov chain Monte Carlo (MCMC) simulations. Trace plots (Figure S3) showed that the chains mixed well and stabilised, suggesting good convergence. Furthermore, the Monte Carlo standard errors (MCSEs) for all coefficient estimates were less than 5% of their corresponding posterior standard deviations (Table 4). This level of precision indicates that the number of MCMC iterations was sufficient for reliable estimation of the posterior distributions.

## 4. Discussion

Our study has found that loneliness and social isolation exhibited spatial variation across Australia, as well as within individual states in Australia. Socio-economic disadvantage (measured by IRSAD) and the proportion of the population with depression and/or anxiety were significantly associated with spatial variation in risk of loneliness, while the average number of hospital admissions at a geographic level was the only factor found to be related to areal relative risk of social isolation.

Interpreted through an opportunity structures’ lens, our findings suggest that socio-economic disadvantage and mental health burden at the area level may reflect constrained local environments that limit opportunities for social participation and support, thereby increasing the risk of loneliness. Conversely, regions with greater structural resources may offer protective social opportunities even in the presence of individual vulnerability.

Our finding that areal measure of socio-economic status (SES) was associated with loneliness is supported by several studies. An American study of 1235 participants from the primary care setting, aggregated at 44 zip code geographies [35], found that poverty, Social Deprivation Index scores, proportions of unemployment, education and lower household income were associated with loneliness. A model that incorporates spatial clustering and includes a larger geographical coverage could improve precision and generalisability in that study. SES was found to be generally non-significant when studying loneliness and social isolation on selected waves from the HILDA database in a recent study [4], but the difference in findings could be due to contextual SES assigned to the individual level and analysed at an individual level, compared to the spatial areal analysis employed in our study.

A non-spatial Danish study [36] found residents of deprived neighbourhoods had significantly higher odds of loneliness, and also that these groups had a high risk of co-occurrence of health-risk behaviours (e.g., smoking, alcohol, physical inactivity, etc.). A recent non-spatial analysis in Australia [1] also demonstrated a positive association between IRSAD and both episodic and chronic loneliness, along with another study undertaken in a less generalisable and smaller geographical setting (Census Collection District, CCD in Brisbane, Australia) [5]. These studies collectively add strength to the evidence on the relationship between areal SES and loneliness, but gaps lie in the analytical methodologies and generalisability of findings.

The association between area-level SES and loneliness we found may be explained by the contextual social and structural conditions that help shape individual experiences, with areal SES potentially hampering opportunities for meaningful social interaction and support. As highlighted in a recent systematic review [6], socio-economic aspects of neighbourhoods, including areal measures of SES, can contribute to loneliness by limiting access to social capital and fostering social exclusion, particularly when individuals perceive their environment as unsuitable for walking, unsafe or lacking cohesion. This could also be due to a lack of social infrastructure that is important for sharing time, space and activities, which can alleviate social isolation and loneliness.

In terms of health utilisation factors, a related study [1] found that long-term health conditions were associated with both episodic (adjusted odds ratio, AOR = 1.2) as well as chronic (AOR = 2.0) loneliness at the individual level. Possible explanations for why our study differed could be due to our evaluation at the aggregate (contextual) measure of long-term health conditions as opposed to individual-level effects which are quite different concepts. Our study also considered the “burden” of loneliness and social isolation at the geographic level, as quantified by relative risk estimates, rather than defining them as chronic or episodic at the individual level.

In our study, while the proportion of people with any chronic/serious illness diagnoses, depression and anxiety, average number of doctor visits and average number of hospital admissions at the SA3 level were associated with loneliness in the univariable analysis, only SES (IRSAD), depression and anxiety were significant in the final model, indicating possible confounding and highlighting the more important covariates. Our study also did not find a significant association with rurality. Several health geography studies report mixed evidence regarding rurality and loneliness/social isolation. Some work suggests that rural residents do not experience consistently higher loneliness than urban counterparts, with moderating effects by sociodemographic factors observed [37]. These findings align with our results, suggesting that rural/urban differences in loneliness may be mediated by composition and local opportunity structures rather than by rural status alone [38]. Future studies could explore whether rurality’s impact varies by age group, particularly among older adults in rural areas, where social isolation may be more pronounced [39]. Accordingly, our findings should not be interpreted as diminishing the significance of loneliness in rural contexts, but rather as indicating that observed spatial disparities are more plausibly associated with uneven opportunity structures and service accessibility than by rurality per se.

The link between loneliness and mental health has been well reported extensively at the individual level [40]. Our results confirm these associations at the areal level. Several plausible mechanisms may explain the observed area-level associations between loneliness, mental health burden, and healthcare utilisation. Recent empirical evidence suggests that loneliness may contribute indirectly to chronic disease burden through depressive symptoms and subsequent deterioration in self-perceived health, with meaningful heterogeneity by age group [9]. At the population level, regions with a higher prevalence of depression and anxiety may therefore experience a compounded burden of loneliness and downstream health needs, which may manifest as higher healthcare utilisation rather than a direct effect of loneliness alone. The bi-directional relationship between loneliness and mental health has been demonstrated in one study [41], which found that earlier loneliness predicted social anxiety, paranoia and depression. In contrast, our study design is ecological and cross-sectional, so it would be difficult to establish causality in the relationships between variables. Similarly, associations at the areal (contextual) level cannot be extrapolated to the individual level, as these may diverge. For example, an area might show an association between high loneliness and poor mental health, but this does not mean that every lonely individual in that area has poor mental health.

Level of greenness, as measured by NDVI, was not found to be associated with loneliness in our study. Even though we found higher values of NDVI to be protective of social isolation in the univariable models, that association was no longer significant when average number of hospital admissions was included, indicating that the latter was a stronger factor. Our finding that social isolation was significantly associated with the number of hospital admissions warrants further discussion. It has been demonstrated that loneliness (social isolation was not studied) was associated significantly with hospital emergency department use, independent of chronic illness [42], while another review [43] showed social isolation and loneliness were consistently linked to increased health care utilisation, particularly among older adults, which also includes studies showing higher rates of hospital readmissions, longer hospital stays, and more frequent physician visits in those with limited social support. These patterns may be associated with a lack of informal care networks to assist with either early intervention and/or post-discharge support. At the area level, higher hospital admission rates may reflect regions where loneliness or social isolation, mental health burden, and chronic disease co-occur, rather than a direct causal effect of loneliness on hospital use. Reverse causation is also plausible, whereby higher healthcare utilisation reflects underlying morbidity that may itself contribute to social isolation and loneliness.

Loneliness and social isolation showed overlapping but distinct geographic patterns, reflecting their conceptual differences as subjective and objective constructs. Loneliness, as a perceived discrepancy between desired and actual social relationships, is shaped by individual perceptions and psychological context, and is therefore less fully explained by observable area-level socio-demographic characteristics. In contrast, social isolation reflects more objective markers of social contact and network structure, making it more sensitive to the compositional profile of populations within areas. Consistent with this, different sets of area-level factors were associated with each outcome in the multivariable models, with socio-economic disadvantage and mental health burden associated with loneliness, and healthcare utilisation (hospital admissions) associated with social isolation. All fixed and random effects in the Bayesian models were assigned weakly informative priors, which limits the influence of prior assumptions on the posterior estimates and reduces the likelihood that results are driven by prior specification. While this lessens the need for extensive sensitivity analyses, we acknowledge that formal sensitivity testing was not undertaken due to computational constraints, and future studies using high-performance computing or approximate Bayesian approaches could further assess the robustness of model assumptions.

Our rationale for the choice of SA3 as the geographical unit of analysis was based on a trade-off between precision in estimating the relative risks and prevalence of outcomes at small area levels. This unit of analysis is also consistent with how Australian government agencies have presented health data geographically [27,44]. At the other end of the spectrum, spatial analysis at the Census Collection District (CCD) level across Australia (n~39,000) would be ideal, but computationally challenging, potentially identifying individuals and the absolute outcome counts may not be large enough to provide reasonable precision in the relative risk estimates. Government policies and health interventions are also usually targeted at a broader geography level like SA3, as compared to CCD level.

### 4.1. Implications

Our research on the spatial distribution of loneliness and social isolation provides insights into identifying communities most at risk and can assist policymakers, health and community services, and local governments in providing solutions for loneliness in geographical areas where it is most needed, especially in urban settings and regions with low neighbour contact or weak community ties. This evidence-based spatial approach offers an actionable strategy for designing inclusive, place-based programs and establishing and maintaining community infrastructure that can initiate and maintain healthy social connection. This approach can guide equitable resource allocation to support and maintain healthy communities. Health and community services including general practice, allied health providers and community mental health centres can also link individuals to community-based social activities tailored to their interests and cultural backgrounds. A more holistic response and further opportunity for local community to co-design is also critical to ensure engagement with both services and spaces.

The results from our study also provide a meaningful research framework for other researchers. For instance, based on our loneliness and social isolation hot-spots, geographically targeted case–control studies can be conducted by researchers to further elucidate the factors driving the increased risk of loneliness and social isolation in some regions and the lower risk observed in other regions. These studies can include surveys (individual level risk profiles) as well fieldwork on the environment (walkability, facilities, green space, etc.) to provide a targeted approach to understanding the drivers of loneliness and social isolation. Secondly, the geospatial models we have implemented can be used to study the effectiveness of specific targeted community interventions by providing a platform to analyse change in outcomes in a geographical region over time through an appropriate regression model like the one we employed.

### 4.2. Strengths and Weaknesses

The measurement of loneliness and social isolation in our study is based on single-item and categorical indicators, which may not fully capture the multidimensional nature of these constructs. This may shape the observed geographic clustering, along with residential mobility, selective non-response and unmeasured confounders such as local social infrastructure. Second, health conditions, service utilisation (e.g., doctor visits, hospitalisations), and mental health status were self-reported, which may introduce recall bias or misclassification compared to administrative data or formal diagnostic assessments. Our analysis also utilised counts of episodes of loneliness and social isolation across the data collection time periods so as to establish the “burden of loneliness” at each SA3 level.

Adjacency between SA3 regions was also defined based on contiguity rather than land area. We note that this may lead to larger or more highly connected SA3s having a relatively greater influence on spatial model estimates, which should be considered when interpreting the results. As this study is ecological in design, associations observed at the SA3 level cannot be interpreted as individual-level effects, and ecological fallacy [45,46] remains a possibility. We did not undertake multiple imputation to address missing data, as this approach relies on the assumption that data are missing at random (MAR), which may not be appropriate in longitudinal surveys such as HILDA where non-response may be systematically related to factors such as mental health, social disengagement, or socio-economic disadvantage. Future studies incorporating a broader set of auxiliary variables that predict non-response may help support more robust imputation approaches.

Our findings should not be interpreted as causal. The observed associations reflect geographic distribution after adjustment for measured covariates and may be influenced by unmeasured confounding and the cross-sectional nature of the data. The temporal ordering of loneliness, mental health burden, and healthcare utilisation cannot be established, and reverse causation is plausible. Accordingly, findings should be interpreted as area-level patterns suitable for service planning, resource allocation, and hypothesis generation rather than evidence of causal pathways operating at the individual level. We also acknowledge the issue of the modifiable areal unit problem (MAUP) [47], particularly that the magnitude of associations found may differ according to the areal unit of aggregation.

A major strength of this study is the large sample size and comprehensive geographic coverage across the majority of Australia, including metropolitan, regional and remote regions. Health surveys typically suffer from poor response rates; however, that was not the case in our study, with the HILDA data collection achieving a good response rate of 90%+ [48], providing robust data for analysis. This is complemented by the use of sophisticated and appropriate Bayesian spatial models that account for spatial correlation and improve the precision of relative risk estimates of loneliness and SI through “borrowing strength”.

## 5. Conclusions

This study provides the first spatial analysis of loneliness and social isolation in Australia using a Bayesian modelling framework, revealing substantial geographic disparities across SA3 regions. Socioeconomic disadvantage and a higher local burden of depression and anxiety were independently associated with increased relative risk of loneliness, while frequent hospital admissions were associated with social isolation. These findings suggest that place-based contextual factors play a meaningful role in the distribution of loneliness and social isolation, reinforcing the importance of targeting interventions towards communities with socio-economic disadvantage and mental health burden. Our approach demonstrates the value of using spatial statistical techniques to produce more stable and interpretable regional estimates that can support the development of healthy and connected communities, health service planning and policy development. Our findings also offer a framework for future geographically targeted case–control studies and intervention evaluations, useful for evaluating population level impacts. By identifying regions most at risk, we can better inform resource allocation, direct scarce resources, and tailor both universal and targeted solutions to local needs.

## Figures and Tables

**Table 1:** Geographic description of covariates at the SA3 level in Australia (2001 to 2022).

Covariates	Mean	Min	Max
Percentage male	49.6%	46.7%	66.6%
Percentage Australian born	70.6%	32.6%	87.4%
Percentage Aboriginal and Torres Strait Islander	4.7%	0.0%	68.8%
Percentage aged > 65	18.1%	0.0%	40.4%
Percentage with any chronic/serious illness diagnoses	46.5%	0.0%	100.0%
Percentage with depression and/or anxiety	15.4%	0.0%	50.0%
Number of doctor visits (past 12 months)	4.77	1.00	10.96
Percentage with long term health conditions	28.2%	0.0%	76.5%
Number of hospital admissions (past 12 months)	0.21	0.00	0.82
NDVI	0.48	0.16	0.74

Note: SA3: Statistical Area 3, NDVI: Normalised Difference Vegetation Index.

**Table 2:** Univariable factors associated with spatial variation in relative risk of loneliness at SA3 level.

Covariates	RR	SD	MC Error	2.5%	97.5%
IRSAD					
Quintile 1 (highest disadvantaged)	Reference				
Quintile 2	0.948	0.028	0.001	0.897	1.001
Quintile 3	**0.885**	**0.030**	**0.001**	**0.836**	**0.938**
Quintile 4	**0.801**	**0.031**	**0.001**	**0.755**	**0.851**
Quintile 5 (most advantaged)	**0.754**	**0.030**	**0.001**	**0.711**	**0.799**
Percentage aged >65 (every % point increase)	0.947	0.193	0.006	0.636	1.411
Remoteness					
Major cities	Reference				
Regional	1.065	0.034	0.001	0.996	1.135
Remote	1.010	0.064	0.002	0.888	1.154
Percentage male (every % point increase)	1.153	1.204	0.040	0.052	7.668
Percentage Australian born (every % point increase)	1.001	0.111	0.004	0.808	1.272
Percentage Aboriginal and Torres Strait Islanders (every % point increase)	1.609	0.277	0.005	0.958	2.779
NDVI Category					
<0.2	Reference				
0.2–0.4	0.980	0.215	0.007	0.549	1.488
0.4–0.6	1.007	0.214	0.007	0.563	1.513
>0.6 (most green)	0.971	0.216	0.007	0.537	1.468
Walkability Index Category					
Non-metro areas (least walkable)	Reference				
Quartile 1	0.981	0.035	0.001	0.912	1.048
Quartile 2	0.968	0.035	0.001	0.903	1.034
Quartile 3	**0.911**	**0.040**	**0.001**	**0.844**	**0.986**
Quartile 4 (most walkable)	**0.886**	**0.044**	**0.001**	**0.814**	**0.967**
Percentage with any chronic/serious illness diagnoses (every % point increase)	**2.207**	**0.123**	**0.004**	**1.736**	**2.804**
Percentage with depression and/or anxiety (every unit increase)	**6.080**	**0.202**	**0.006**	**4.108**	**9.016**
Number of doctor visits (every % point increase)	**1.081**	**0.009**	**<0.001**	**1.061**	**1.100**
Percentage with long term health conditions (every % point increase)	0.145	3.073	0.122	0.001	3.297
Number of hospital admissions (every unit increase)	**1.680**	**0.137**	**0.004**	**1.275**	**2.186**

Note: SA3: Statistical Area 3, RR: relative risk, SD: standard deviation, MC: Monte Carlo, IRSAD: Index of Relative Socio-economic Advantage and Disadvantage, NDVI: Normalised Difference Vegetation Index. Figures in the table in bold indicate statistically significant data.

**Table 3:** Univariable factors associated with spatial variation in relative risk of social isolation at SA3 level.

Covariates	RR	SD	MC Error	2.5%	97.5%
IRSAD					
Quintile 1 (highest disadvantaged)	Reference				
Quintile 2	0.887	0.080	0.003	0.754	1.027
Quintile 3	0.848	0.088	0.003	0.712	1.003
Quintile 4	**0.831**	**0.088**	**0.003**	**0.700**	**0.986**
Quintile 5 (most advantaged)	0.861	0.085	0.003	0.728	1.010
Percentage aged >65 (every % point increase)	1.688	0.493	0.015	0.733	4.909
Remoteness					
Major cities	Reference				
Regional	**1.185**	**0.067**	**0.002**	**1.039**	**1.355**
Remote	1.161	0.145	0.004	0.889	1.549
Percentage male (every % point increase)	1.249	1.481	0.049	0.050	31.881
Percentage Australian born (every % point increase)	1.036	0.231	0.007	0.654	1.693
Percentage Aboriginal and Torres Strait Islanders (every % point increase)	2.056	0.517	0.012	0.799	5.669
NDVI Category					
<0.2	Reference				
0.2–0.4	1.047	0.614	0.020	0.526	9.728
0.4–0.6	1.100	0.612	0.020	0.547	10.145
>0.6	1.062	0.616	0.020	0.526	10.237
Walkability Index Category					
Non-metro areas	Reference				
Quartile 1 (least walkable)	**0.822**	**0.084**	**0.002**	**0.699**	**0.970**
Quartile 2	**0.798**	**0.084**	**0.002**	**0.681**	**0.947**
Quartile 3	0.853	0.087	0.002	0.721	1.006
Quartile 4 (most walkable)	1.115	0.091	0.002	0.947	1.341
Percentage with any chronic/serious illness diagnoses (every % point increase)	1.580	0.323	0.011	0.899	3.059
Percentage with depression and/or anxiety (every % point increase)	**3.096**	**0.519**	**0.016**	**1.088**	**8.688**
Number of doctor visits (every unit increase)	**1.069**	**0.026**	**0.001**	**1.014**	**1.122**
Percentage with long term health conditions (every % point increase)	**1.210**	**0.113**	**0.004**	**1.012**	**1.525**
Number of hospital admissions (every unit increase)	**3.688**	**0.297**	**0.008**	**2.056**	**6.567**

Note: SA3: Statistical Area 3, RR: relative risk, SD: standard deviation, MC: Monte Carlo, IRSAD: Index of Relative Socio-economic Advantage and Disadvantage, NDVI: Normalised Difference Vegetation Index. Figures in the table in bold indicate statistically significant data.

**Table 4:** Multivariable factors associated with spatial variation in relative risk of loneliness and social isolation.

Covariates	RR	SD	MC Error	2.5%	97.5%	RR	SD	MC Error	2.5%	97.5%
Loneliness						Social Isolation				
IRSAD										
Quintile 1 (highest disadvantaged)	Reference									
Quintile 2	0.971	0.026	0.001	0.923	1.019					
Quintile 3	**0.908**	**0.028**	**0.001**	**0.858**	**0.958**					
Quintile 4	**0.840**	**0.029**	**0.001**	**0.794**	**0.888**					
Quintile 5 (most advantaged)	**0.800**	**0.029**	**0.001**	**0.755**	**0.846**					
Percentage with depression and anxiety	**4.263**	**0.187**	**0.004**	**2.962**	**6.135**					
Number of hospital admissions						**3.688**	**0.297**	**0.008**	**2.056**	**6.567**

Note: RR: relative risk, SD: standard deviation, MC: Monte Carlo, IRSAD: Index of Relative Socio-economic Advantage and Disadvantage, NDVI: Normalised Difference Vegetation Index. Figures in the table in bold indicate statistically significant data.

## Data Availability

This study uses unit record data from the Household, Income and Labour Dynamics in Australia (H.I.L.D.A) Survey, conducted by the Australian Government Department of Social Services (DSS). The findings and views expressed in this paper are solely those of the author(s) and are not attributable to the Australian Government, DSS, or any of its contractors or partners. https://dataverse.ada.edu.au/dataset.xhtml?persistentId=doi:10.26193/R4IN30 (accessed on 27 August 2024). Access to the data is available upon application to the data custodian via the Australian Data Archive.

## Supplementary Materials

The following supporting information can be downloaded at: https://www.mdpi.com/article/10.3390/healthcare14101318/s1, Figure S1. Distribution of relative risk of loneliness across Australia (SA3). Figure S2. Distribution of relative risk of social isolation across Australia (SA3). Figure S3. Diagnostics for final multivariable model. Table S1. Provenance and harmonisation of covariates. Table S2. Partitioning random effects into spatially structured and spatially unstructured effects.
