# Supplementary material for: Geographic Variation in Loneliness and Social Isolation in Australia: Socio-Demographic and Healthcare Utilisation Determinants
Source: Healthcare (Basel). 2026 May 12;14(10):1318. doi: 10.3390/healthcare14101318 (PMC13205294; doi:10.3390/healthcare14101318)
Supplement: Supplementary file 1 [file healthcare-14-01318-s001.zip › healthcare-4247214-supplementary.pdf]

Supplementary data

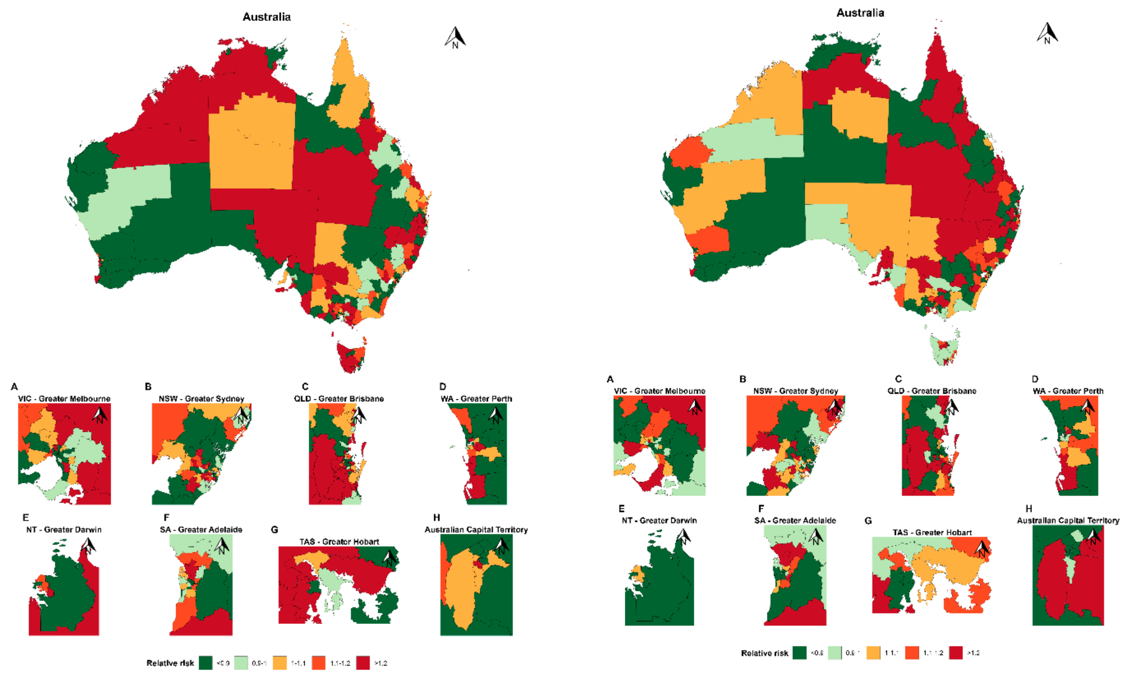

(a) 2001-2007 (b) 2008-2014

Figure S1 shows the spatial distribution of the relative risk of loneliness across Australian SA3 regions for two earlier study periods (2001–2007 and 2008–2014). The maps highlight geographic clustering, where some regions consistently demonstrate higher or lower risk compared with the national average, allowing comparison of whether loneliness hotspots persisted or shifted over time.

Figure S1. Distribution of relative risk of loneliness across Australia (SA3)

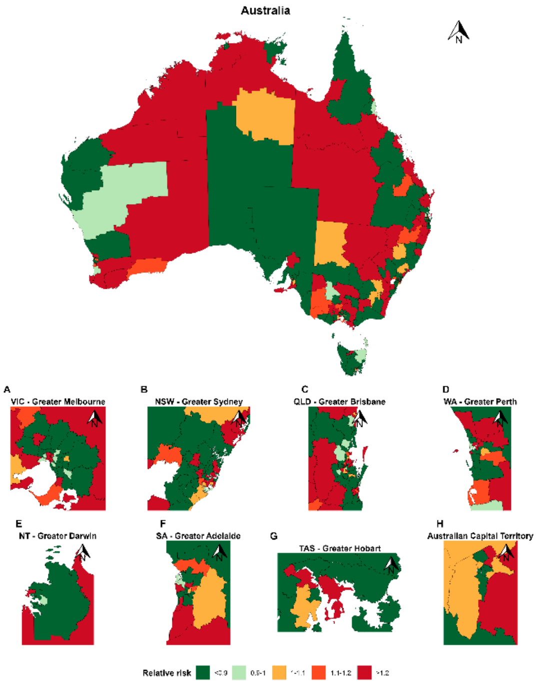

(a) 2001-2007

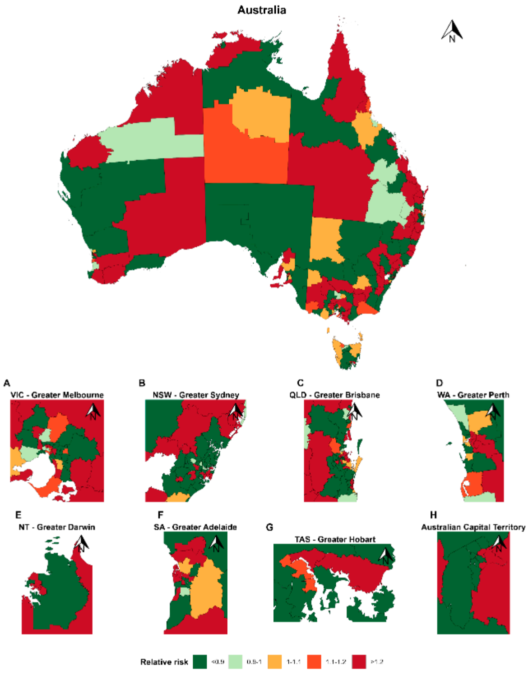

(b) 2008-2014

Figure S2 presents the corresponding spatial distribution of the relative risk of social isolation across SA3 regions for the same two periods (2001–2007 and 2008–2014). By comparing these maps across time, the figures illustrate the stability or change in regional patterns of social isolation and help contextualise the more recent estimates presented in the main paper (2015–2022).

Figure S2. Distribution of relative risk of social isolation across Australia (SA3)

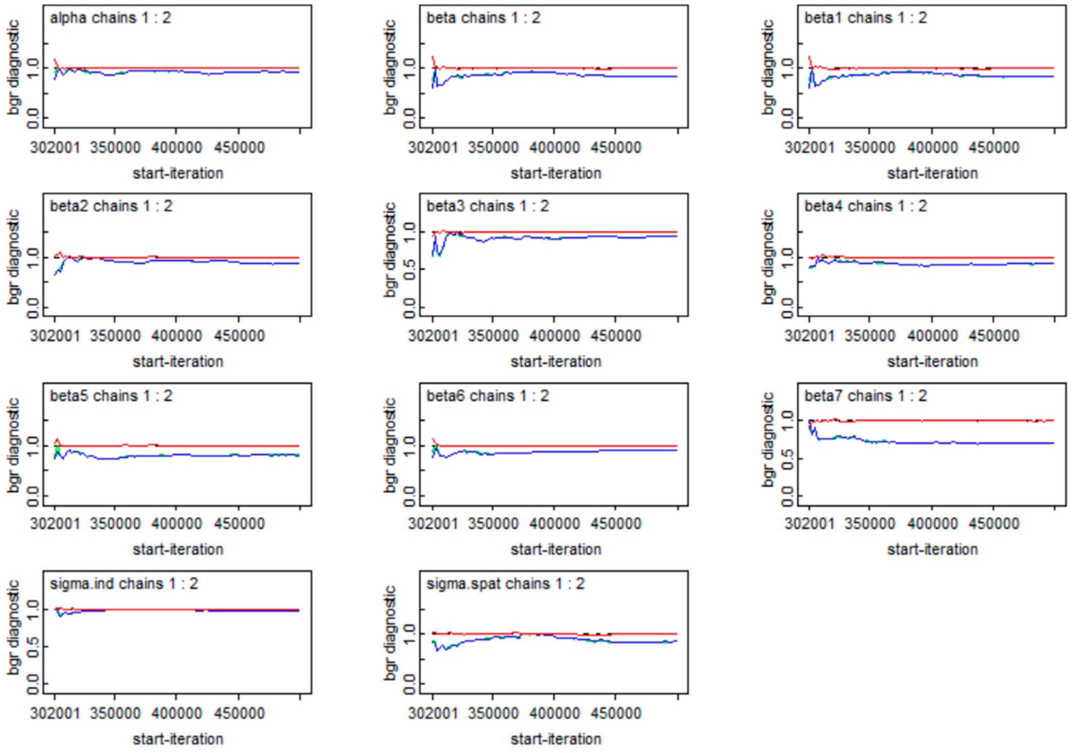

(a) <u>Brooks Gelman Rubin diagnostics plots (Loneliness)</u>

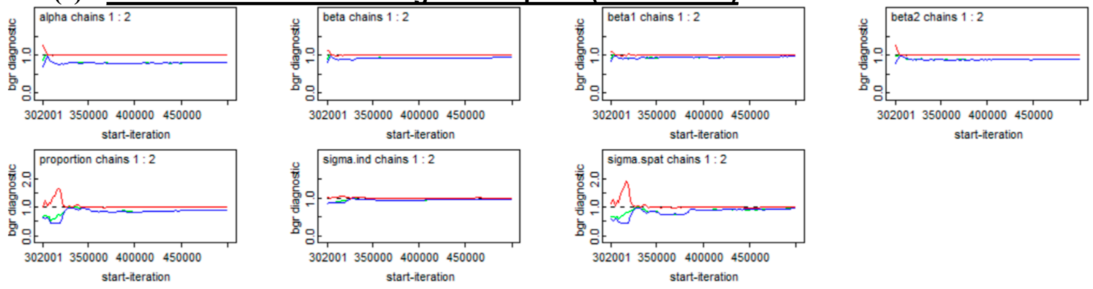

(b) <u>Brooks Gelman Rubin diagnostics plots (Social Isolation)</u>

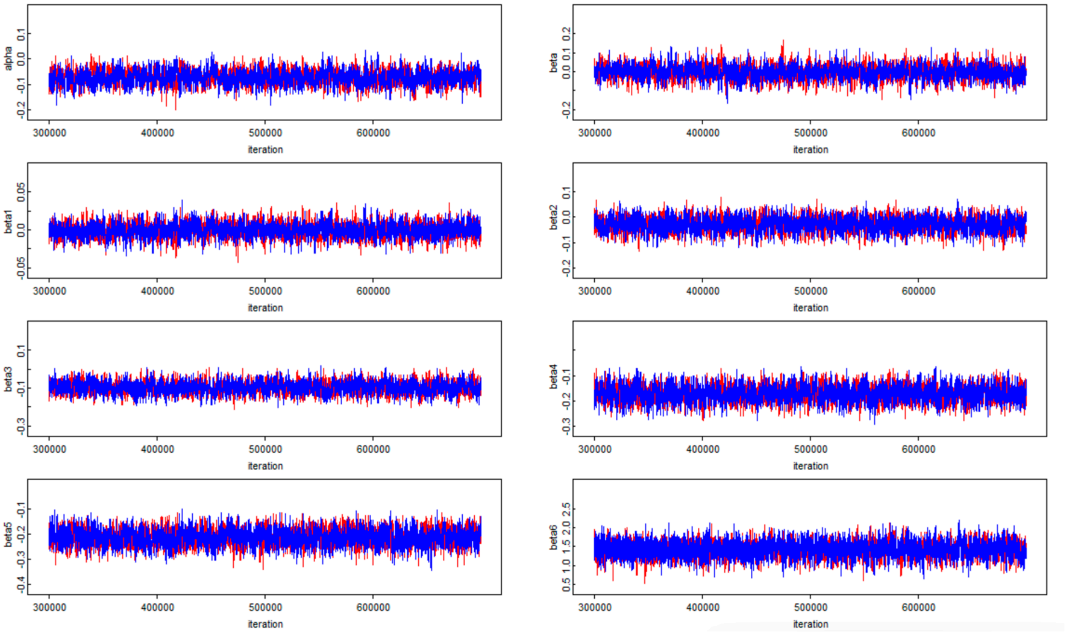

(c) Trace plots (Loneliness)

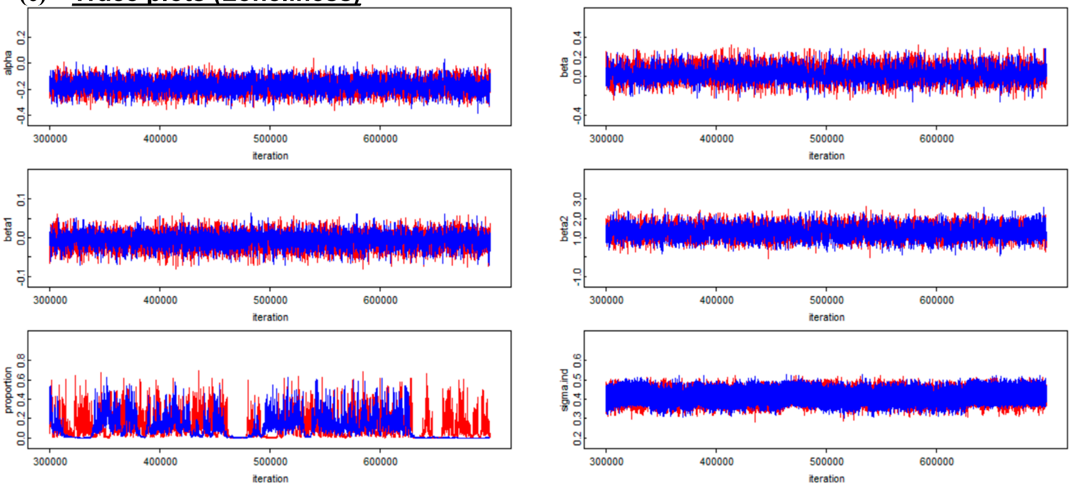

(d) Trace plots (Social Isolation)

Figure S3. Diagnostics for final multivariable model

Table S1. Provenance and harmonisation of covariates

Covariates	Source	Spatial resolution	Reference year(s)	Harmonisation	Variable definition
Loneliness & SI & Healthcare utilisation	HILDA	Individual to SA3	2001–2022	Aggregated by SA3-period	Loneliness and social isolation: derived binary variables at the individual level. Summed up to SA3/ survey period to provide observed, expected counts and relative risk estimates. Number of doctor visits and hospital admissions in the past year (mean calculated at SA3 level), numbers with any chronic/ serious illness diagnoses lasting 6 months or more, and self-reported diagnosis of depression or anxiety, aggregated to proportion at the SA3 level.

IRSAD, remoteness and demographics	ABS	SA3	2021	Applied as time-invariant	IRSAD: Initial scores standardised to mean 1000 (SD=100), were then grouped into quintiles. Remoteness: major cities, inner regional and outer regional grouped, remote and very remote grouped. Demographics: numbers of males, aged >65, ATSI and Australian born, aggregated to proportion at the SA3 level.
NDVI	MODIS MOD13 Q1	250 m	Long-term average	Area-weighted to SA3	Original NDVI ranging from −1 to +1; higher values indicate denser green vegetation. Long-term mean computed across available satellite observations.
Walkability	ANLD	SA3	2018	Time-invariant, quartiles	Index of neighbourhood walkability derived from land use mix, street connectivity, and residential density. Raw standardised score grouped into quartiles and non-metro areas.

Notes: SI: Social Isolation, HILDA: Household, Income and Labour Dynamics in Australia, SA3: Statistical Area 3, IRSAD: Index of Relative Socio-economic Advantage and Disadvantage, ABS: Australian Bureau of Statistics, ATSI: Aboriginal and Torres Strait Islander, NDVI: Normalized Difference Vegetation Index, MODIS: Moderate Resolution Imaging Spectroradiometer, MOD13: MODIS Terra land vegetation index product (Vegetation Indices), ANLD: Australian National Liveability Dataset.

Table S2. Partitioning random effects into spatially structured and spatially unstructured effects

**Loneliness**	**Mean**	**SD**	**MC Error**	**2.5%**	**97.5%**
Proportion of total variation attributed to spatial effects	0.582	0.125	0.003	0.323	0.804
Spatially unstructured SD	0.135	0.014	0.000	0.106	0.162
Spatially structured SD	0.163	0.030	0.001	0.107	0.224
**Social Isolation**					
Proportion of total variation attributed to spatial effects	0.147	0.097	0.003	0.015	0.374
Spatially unstructured SD	0.422	0.025	0.000	0.374	0.471
Spatially structured SD	0.167	0.066	0.002	0.054	0.305

Note: SD: Standard Deviation, MC: Monte Carlo.
